# Supplementary material for: Why do people sell their kidneys? A thematic synthesis of qualitative evidence
Source: PLOS Glob Public Health. 2024 Mar 27;4(3):e0003015. doi: 10.1371/journal.pgph.0003015 (PMC10971689; doi:10.1371/journal.pgph.0003015)
Supplement: S1 Table — (DOCX) [file pgph.0003015.s002.docx]

**S1 Table**

In MEDLINE (PubMed):

("kidney"[MeSH Terms] OR "kidney"[All Fields] OR "kidneys"[All Fields] OR "kidney s"[All Fields] OR "commerce"[MeSH Terms] OR "commerce"[All Fields] "Deal"[MeSH Terms] OR "Deal"[All Fields] OR "sale"[All Fields] OR "commerce"[MeSH Terms] OR "commerce"[All Fields] OR "commerce"[All Fields] OR "trade"[All Fields] OR "traded"[All Fields] OR "trades"[All Fields] OR "trading"[All Fields]) AND (1987:2024[pdat])

In Scopus (Elsevier) and JSTOR,

we searched using “kidney” [AND] “sale” [OR] “trade” [OR] “deal” [OR] “Commerce” in the advanced section.
