# Supplementary material for: Why do people sell their kidneys? A thematic synthesis of qualitative evidence
Source: PLOS Glob Public Health. 2024 Mar 27;4(3):e0003015. doi: 10.1371/journal.pgph.0003015 (PMC10971689; doi:10.1371/journal.pgph.0003015)
Supplement: S2 Table — (DOCX) [file pgph.0003015.s003.docx]

**S2 Table. Comprehensiveness of reporting of included studies (COREQ)**

| **Items** | | **Included studies** | | | | | | | | | | | | | | |
| --- | --- | --- | --- | --- | --- | --- | --- | --- | --- | --- | --- | --- | --- | --- | --- | --- |
|  |  | **Lawrence Cohen -1999** | Nancy Scheper-Hughes  2000 | Javaad Zargooshi  2001 | Diane M. Tober  2007 | Farhat Moazam  2009 | Awaya Tsuyoshi  2009 | Roger Lee Mendoza  2010 | Susanne Lundin  2012 | Monir Moniruzzaman  2012 | Medel Salvador Paguirigan  2012 | Farhan Navid Yousaf  2015 | Monir Moniruzzaman  2016 | Frederike Ambagtsheer  2019 | **(**  **WWidodo**  **2021** | **x**  BBijaya Shrestha  22022 |
| **Domain 1: Research team and reflexivity** | |  |  |  |  |  |  |  |  |  |  |  |  |  |  |  |
| ***Personal characteristics*** | |  |  |  |  |  |  |  |  |  |  |  |  |  |  |  |
| 1 | Interviewer/facilitator | *✓* | *-* | *-* | *-* | *✓* | *-* | *-* | *✓* | *✓* | *✓* | *✓* | *✓* | - | -- | *✓✓* |
| 2 | Credentials | *✓* | *-* | *-* | *-* | *✓* | - | - | - | *✓* | *✓* | *✓* | *✓* | - | -- | *✓✓* |
| 3 | Occupation | - | *-* | *-* | *-* | *✓* | *-* | *-* | *✓* | *✓* | *✓* | *✓* | *✓* | - | -- | *✓✓* |
| 4 | Gender | - | *-* | *-* | *-* | *✓* | - | - | - | *✓* | - | - | *✓* | - | -- | -*-* |
| 5 | Experience and training | - | *-* | *-* | *-* | *✓* | - | - | - | *✓* | *✓* | *✓* | - | - | -  -- | *✓✓* |
| ***Relationship with participants*** | |  |  |  |  |  |  |  |  |  |  |  |  |  |  |  |
| 6 | Relationship established | *✓* | *-* | *-* | - | - | - | N/A | - | *✓* | *✓* | - | *-* | *✓* | -- | *✓✓* |
| 7 | Participant knowledge of the interviewer | - | *-* | *-* | *-* | *✓* | - | N/A | - | *✓* | *✓* | - | - | - | -- | *✓✓* |
| 8 | Interviewer characteristics | *✓* | *✓* | *-* | *-* | *✓* | - | N/A | - | *✓* | *✓* | - | - | - | -- | *✓✓* |
| **Domain 2: Study design** | |  |  |  |  |  |  |  |  |  |  |  |  |  |  |  |
| ***Theoretical framework*** | |  |  |  |  |  |  |  |  |  |  |  |  |  |  |  |
| 9 | Methodological orientation and Theory | *✓* | - | *✓* | *✓* | - | *✓* | *-* | *✓* | *-* | *✓* | *✓* | *-* | *-* | *✓✓* | *✓✓* |
| ***Participant selection*** | |  |  |  |  |  |  |  |  |  |  |  |  |  |  |  |
| 10 | Sampling | - | *✓* | *-* | *-* | *✓* | *✓* | *✓* | - | *✓* | *✓* | *✓* | - | - | -- | *✓✓* |
| 11 | Method of approach | *✓* | *✓* | *✓* | *✓* | *✓* | *✓* | *✓* | *✓* | *✓* | *✓* | *✓* | *-* | *✓* | *✓✓* | *✓✓* |
| 12 | Sample size | - | *✓* | *-* | *-* | *✓* | *✓* | *✓* | - | *✓* | *✓* | *✓* | *✓* | *✓* | *--* | *✓✓* |
| 13 | Non-participation | - | *-* | *-* | *-* | *✓* | - | N/A | - | *✓* | *✓* | - | - | - | -- | -- |
| ***Setting*** | |  |  |  |  |  |  |  |  |  |  |  |  |  |  |  |
| 14 | Setting of data collection | *✓* | *✓* | *✓* |  | *✓* | *✓* | N/A | *✓* | *✓* | *✓* | *✓* | *-* | *✓* | *--* | *✓✓* |
| 15 | Presence of non- participants | - | *-* | *✓* |  | *✓* | - | N/A | - | *✓* | *✓* | - | *-* | *✓* | *--* | *--* |
| 16 | Description of sample | *✓* | *✓* | *-* |  | *✓* | *✓* | *✓* | *✓* | *✓* | *✓* | *✓* | *✓* | - | -- | *✓✓* |
| ***Data collection*** | |  |  |  |  |  |  |  |  |  |  |  |  |  | - |  |
| 17 | Interview guide | - | *✓* | - |  | *✓* | *✓* | N/A | - | - | *✓* | - | *-* | *✓* | *--* | *✓✓* |
| 18 | Repeat interviews | - | - | - |  | *✓* | - | N/A | - | - | *✓* | - | - | - | -- | *✓✓* |
| 19 | Audio/visual recording | *✓* | *✓* | - |  | *✓* | - | N/A | - | - | *✓* | - | *-* | *✓* | *--* | *✓✓* |
| 20 | Field notes | - | - | - |  | *✓* | - | N/A | - | - | - | - | - | - | -- | *✓✓* |
| 21 | Duration | - | *✓* | *✓* |  | *✓* | *✓* | N/A | - | *✓* | *✓* | *✓* | *-* | *✓* | *--* | *✓✓* |
| 22 | Data saturation | - | *✓* | - |  | *✓* | - | N/A | - | *✓* | *✓* | - | - | - | -- | -- |
| 23 | Transcripts returned | - | *-* | *-* |  | - | - | N/A | - | *✓* | *✓* | - | - | - | -- | -- |
| 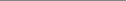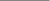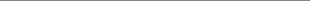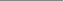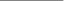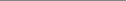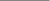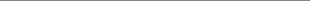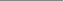  ***Data analysis*** | |  |  |  |  |  |  |  |  |  |  |  |  |  |  |  |
| 24 | Number of data coders | - | - | - | - | - | - | - | - | - | - | - | - | - | -- | *✓✓* |
| 25 | Description of the coding tree | - | - | - | - | - | - | - | - | - | - | - | *-* | *✓* | *--* | *--* |
| 26 | Derivation of themes | - | - | *✓* | *✓* | *✓* | *✓* | *✓* | - | *✓* | *✓* | - | *-* | *✓* | *✓✓* | *✓✓* |
| 27 | Software | - | - | - | - | - | - | - | - | - | - | - | *-* | *✓* | *--* | *✓✓* |
| 28 | Participant checking | - | - | *-* | - | - | - | - | - | *✓* | - | - | - | - | -- | -- |
| ***Reporting*** | |  |  |  |  |  |  |  |  |  |  |  |  |  |  |  |
| 29 | Quotations presented | *✓* | *✓* | *✓* | *✓* | *✓* | *✓* | *-* | *✓* | *✓* | *✓* | *✓* | *-* | *✓* | *--* | *--* |
| 30 | Data and findings consistent | - | - | *✓* | *✓* | *✓* | *✓* | - | - | *✓* | *✓* | *✓* | *-* | *✓* | *✓✓* | *✓✓* |
| 31 | Clarity of major themes | *✓* | - | *✓* | *✓* | *✓* | *✓* | *✓* | *✓* | *✓* | *✓* | *✓* | *-* | *✓* | *✓✓* | *✓✓* |
| 32 | Clarity of minor themes | - | - | *✓* | *✓* | *✓* | *✓* | - | - | - | *✓* | - | - | *✓* | *✓✓* | *✓✓* |

*Note: (✓) indicates reported by the study, (– ) indicates not stated in the study, and (N/A) denotes not applicable in the study.*
