# Supplementary material for: Why do people sell their kidneys? A thematic synthesis of qualitative evidence
Source: PLOS Glob Public Health. 2024 Mar 27;4(3):e0003015. doi: 10.1371/journal.pgph.0003015 (PMC10971689; doi:10.1371/journal.pgph.0003015)
Supplement: S3 Table — (DOCX) [file pgph.0003015.s004.docx]

**S3 Table: Summary of the included studies**

|  | **First Author** | **Year** | **Country** | **Duration of Data collection** | **Methods of Data collection** |
| --- | --- | --- | --- | --- | --- |
| 1 | Lawrence Cohen | 1999 | India | June 1998 (only mentioned) | One and one interview through interpreter. |
| 2 | Nancy Scheper-Hughes | 2000 | Brazil, South Africa, and India. | 1996 and 1998 (6 to 8 weeks) | Multi-sited ethnography with observation, interviews. |
| 3 | Javaad Zargooshi | 2001 | Iran | 2000 | Interview with videotape |
| 4 | Diane M. Tober | 2007 | Iran | 2002 | Qualitative Pilot Investigation |
| 5 | Farhat Moazam | 2009 | Pakistan | 2007 (multiple visit each lasting for 4 to 5 days) | Ethnographic study with interview. |
| 6 | Awaya Tsuyoshi | 2009 | The Philippines | May 2007 to March 2008 | Interview |
| 7 | Roger Lee Mendoza | 2010 | The Philippines | 2008 | Comparative content analysis |
| 8 | Susanne Lundin | 2012 | Moldova and Israel | Mentioned only two trips during 2008 | Ethnographic study with observation and interview |
| 9 | Monir Moniruzzaman | 2012 | Bangladesh | 2005 | In-depth interview. |
| 10 | Medel Salvador Paguirigan | 2012 | The Philippines | December 2010. | Interview. (modified liner snowball sampling) |
| 11 | Farhan Navid Yousaf | 2015 | Pakistan | 2013 to 2014 | Ethnographic study |
| 12 | Monir Moniruzzaman | 2016 | Bangladesh and Canada | Bangladesh (2012 from other study), Canada no reference | Case study |
| 13 | Frederike Ambagtsheer | 2019 | The Netherland | 2011 to 2015 | Qualitative interview study |
| 14 | Widodo | 2021 | Indonesia | 2015 to 2021 | In-depth Interview |
| 15 | Bijaya Shrestha | 2022 | Nepal | 2020 | Ethnography |
