# Supplementary material for: Why do people sell their kidneys? A thematic synthesis of qualitative evidence
Source: PLOS Glob Public Health. 2024 Mar 27;4(3):e0003015. doi: 10.1371/journal.pgph.0003015 (PMC10971689; doi:10.1371/journal.pgph.0003015)
Supplement: S4 Table — (DOCX) [file pgph.0003015.s005.docx]

**S4 Table:** Thematic findings from the studies

| **Level of analysis** | **Key Themes** | **Quotations from participants** | **Interpretation** | **References** |
| --- | --- | --- | --- | --- |
| Individual | Desperations | - “I had to repay a loan of Rs. 165,000 [US$1650]. Despite working so hard I realized I could not repay the loan ... Here one other person had done this before ... Two years ago, I sold my kidney ... They [agent and doctor] had promised me Rs. 200,000 [US$2000], but I received only Rs. 180,000 [US$1800] after deduction of food expenditure during my stay in the hospital ... I had to take loan again when my wife was about to deliver a baby, then my child fell ill and needed blood bottles, and then my mother fell ill. Now again my loan has amounted to Rs. 85,000 [US$850]. I, my wife, and children work in the brick kiln. If I feel ok then we earn 300–350 [US$3–US$3.5] daily.” |  | Farhan Navid Yousaf (2015) |
|  |  | Kidney vending destructed my life. A loan shark lent us money at exorbitant rates of interest and we will never rescue from him. |  | Diane M. Tober (2007) |
|  | Poverty | “Here people are poor, some have to marry their daughters off ... they work at brick kilns. They work in such a hot weather but they don’t receive the wages they deserve. People remain hungry, some fall ill. As people came to know about selling [a] kidney, they presented themselves. Here people don’t have sufficient land for agriculture ... and there is no business. They are all uneducated and ignorant and know nothing. They take loans ... if they don’t work they don’t get food. If they are in tension and there is no solution then people have to do something for their children. They sacrifice their bodies for their children. People do it due to poverty; nobody does it happily. All the men had to repay loans ... their wives are abused and humiliated, they can’t go anywhere ... they can do anything to protect their honor ...” | While the principles  of charity and altruism are invoked, other Islamic principles, for example not  harming one’s body, are explained away: it is OK to harm one’s body in order to save the life of another. | Lawrence Cohen (1999) |
|  |  | We didn’t have a house. We stayed on the streets. We slept there, under a truck. When it rains, we go to the “barangay” [village] hall. We slept on the cold cement.” |  | Medel Salvador Paguirigan (2012) |
|  | Isolation | One of the most devastating effects of vending was damage to social relationships, with 70% who became isolated, irritable and hated social contacts. Many vendors had been so sensitized and felt humiliated when someone talked about any general topic, such as kidney or dialysis. |  | Javaad Zargooshi (2001) |
|  |  |  | The rapid urbanization of Metro Manila, rising poverty level, and lack of job opportunities in the rural areas result in rapid urban population growth due to migration. This rapid population growth resulted in a rapid influx of informal settlers and building shanties that Filipinos call “squatters” (Ibon Foundation, 2010). This form of self-constructed substandard housing the participants built is vulnerable to natural and human- induced catastrophes because the structures are made of light and highly combustible, second-hand materials (plywood, tarp, galvanized iron, and old advertising billboards). Residents live in abject poverty and under squalid conditions. The proximity of the homes to each other makes the entire community susceptible to fire. | Susanne Lundin (2012) |
|  | Egoism | -“He needed a kidney, too,” she says. “He was dying, and received one from his niece; they did the operation in America. At that time, I did not know about kidneys. If I had, I would have given him both of mine.” | While the principles  of charity and altruism are invoked, other Islamic principles, for example not  harming one’s body, are explained away: it is OK to harm one’s body in order to save the life of another. | Lawrence Cohen (1999) |
|  |  | I decided to donate a kidney so that at least two of my children would be able to go to school. I have five children... I was able to provide my family with a home and send my kids to school using that money. It’s very important that I sacrificed my kidney because I was able to help my fel- low man, whose life was extended because of my kidney. I helped them; they also helped me. | Although some participants’ motivation to donate their kidney was to “extend the life” of the recipient, their altruistic intent has been tainted by the act of “trading” their kidneys for money. | Medel Salvador Paguirigan (2012) |
|  | Autonomy | the decision of whether or not to sell one’s organs is ultimately left with the individual, with the government as only the facilitator for the transaction. This notion of bodily autonomy is in direct conflict with most Sunni positions, that the body is owned by God and one does not have authority to sell parts of it. |  | Awaya Tsuyoshi (2009) |
|  | Rational |  | Sellers compare the money made from other jobs and money made by other sellers. | Widodo (2021) |
|  | Threat | We wanted to take the passports and run home from the place. One ran away without his passport. I went back to my employer and said, “If you’ll pay, it’s okay. If not – just give me my passport and I’ll go home.” But I was told that if I refuse to give my kidney, I would be killed – shot. After that I was brought to a hospital, and then they did the surgery. |  | Monir Moniruzzaman (2012) |
| Societal Level | Gender role | “My husband needs strength for work and could not work if he had the operation” |  | Lawrence Cohen (1999) |
|  |  | -   “It is a matter of shame for us [men] if our women get naked [for organ removal].” |  | Farhan Navid Yousaf (2015) |
|  | Social Responsibility | “Here people are poor, some have to marry their daughters off ... they work at brick kilns. They work in such a hot weather but they don’t receive the wages they deserve. People remain hungry, some fall ill. As people came to know about selling [a] kidney, they presented themselves. Here people don’t have sufficient land for agriculture ... and there is no business. They are all uneducated and ignorant and know nothing. “ |  | Farhan Navid Yousaf (2015) |
|  | Family Responsibility | -   He supports his wife and 5 children. “I wanted them to live. I sacrificed my kidney for the two of them [the two sick children],” he said. |  | Medel Salvador Paguirigan (2012) |
|  |  | -   “I sold my kidney ... I had to repay a loan of Rs. 80,000 [US$800] of my sister ... Due to the outstanding loan, brick kiln owner was going to sell my sister. He said he will receive money from the buyer after selling her ... She asked me to help her ... I sold my kidney to help her ... I also took a loan to marry off my daughter.” |  | Monir Moniruzzaman (2016) |
|  |  | “I would do exactly as I said, and I have not regretted my offer. I know that I would have to undergo an operation that is difficult and risky. But I would sell any organ that would not immediately cause my death. It could be a kidney or an eye because I have two of them. . . . I am living through all sorts of crises and I cannot make ends meet. If I could sell a kidney or an eye for that much money I would never have to work again. But I am not stupid. I would make the doctor examine me first and then pay me the money up front before the operation. And after my bills were paid, I would invest what remains in the stock market.” |  | Nancy Scheper-Hughes (2000) |
|  |  |  | In some cases, vendors had borrowed small amounts of money from family members, mainly brothers. The brothers then pressed them to sell their kidneys to pay the debt. In fact, in several cases the sole reason for vending was pressure from the brother. | Javaad Zargooshi (2001) |
|  | Influence of brokers |  | Kidneys are sold due to ignorance and lack of education about the purpose of kidneys. | Lawrence Cohen (1999) |
|  |  |  | Sellers were lured to sell their kidney in huge amount as the basic salary was way less than offered to them. | Widodo (2021) |
|  |  |  | The middleman is in a position to withhold payment from the organ donor and use such payment as an undue inducement. More importantly, middlemen do not have a clear responsibility to protect the interests of donors. | Awaya Tsuyoshi (2009) |
|  |  | If the sellers do not match with the receivers, brokers will try to find new receivers. But if it is matched, then the brokers start to negotiate with the buyers. Brokers are so smart they have started to read the medical situation, psychology, economic conditions of the buyers and they increase the demand accordingly. These brokers have good observation skills, they are street-smart, and they can read and observe the patients. |  | Bijaya Shrestha (2022) |
