# Supplementary material for: Why do people sell their kidneys? A thematic synthesis of qualitative evidence
Source: PLOS Glob Public Health. 2024 Mar 27;4(3):e0003015. doi: 10.1371/journal.pgph.0003015 (PMC10971689; doi:10.1371/journal.pgph.0003015)
Supplement: Attachment — Submitted filename: review.pdf [file pgph.0003015.s006.pdf]

## review

I greatly enjoyed reading the manuscript, which sheds light on a relevant public health issue that requires being properly addressed by authorities worldwide. Appendices are very rich in information.

Some comments and suggestions:

- Row 39 - the notion of goods for pleasure. I would suggest thinking about the notion as not necessarily reflects the purpose of the acquired good. For example, a mobile phone or a motorbike could be an economic means. People might consider it as a "necessity" and not as something superfluous.
- Row 41 - the word "Ironically" doesn't express, at least in my opinion, the tragedy of doing that.
- The introduction does not clearly state the unethical aspect of selling organs. For example, row 75 mentions "policies guiding kidney selling". Is it legal in any country? I understand Iran is the only country that allows it. WHO has issued different condemnations of the financial transactions of organs. For instance, the 44th World Health Assembly.
- Rows 100-102: I would add there are risks of acquiring infectious diseases such as Hepatitis and HIV.
- The readers will benefit from a clear definition of kidney selling in the Introduction. I mention that because the introduction reviews many different modalities (donation from living and death). Is kidney selling equivalent to kidney trafficking? How it differs?
- On the PRISMA graph, it says "Records excluded through title". However, in the text, it explains that the initial screening goes beyond that. I would suggest changing it to "Records excluded through initial screening".
- On the analysis of the comprehensiveness of reporting, I wonder if there should be a discussion or comparison between those who report few items and those who report more items. Does it matter? Does it reflect quality?
- About lens: In the Data synthesis and analysis (row 164ss) says "The thematic analysis took a critical approach to understand the reasons for kidney selling by examining power disparities and social inequalities" and later "We used the critical medical anthropology (CMA) theoretical lens". On the results it mentions the "lens of power relations and social inequities", and on the abstract "The thematic analysis involved a critical approach to understanding". I am a bit confused if there are differences between CMA and critical approach and power relations and social inequalities.
- Results:

  - individual level: "poverty was the major reason for kidney" and "desperation to alleviate poverty" seem similar to me.
  - "Many participants sold their kidneys due to lack of better economic conditions". Do you mean lack of economic means such as jobs or regular income? If not, what is the difference between "poverty" and "lack of better economic conditions"?
  - I think these paragraphs will benefit the reader if they are better organised. I have tried to identify the main topics per paragraph and it becomes difficult because it looks repetitive. I am sure the information is quite rich and relevant. So, for example (you don't have to follow it), organise the paragraphs around topics: poverty, payment of debts, buying goods, altruism, etc.
  - The authors also report some consequences of selling kidneys such as "no economic freedom, poor health, etc". That should go in a different paragraph to make the reading experience clear.

- The same comment about organising the information applies to the meso and macro levels.

- Societal level:

  - explain social conformism;
  - "Some of the participants sold their kidneys due to ignorance and unawareness regarding the function of kidneys". This seems to belong to the individual level. I understand that brokers use that, but it would also be good to highlight it in the individual-level section.
  - The section is called "Societal level" and on row 11 it mentions "intermediate and macro level". Is this a different section?
  - Figure 3 is the author's own. Which author? If this is your contribution, it should be included as a section and explained as a synthesis of the reviews or similar. If you could map back to the original papers that would give robustness to the theoretical contribution.

- Discussion:

  - I find difficult to distinguish between the results and the discussion sections. You could benefit from this "https://plos.org/resource/how-to-write-conclusions/"
  - The fact that Iran has a "legal" structure of selling kidneys should be accounted for in the analysis, for example, to compare with other countries in the discussion.
  - Mentions "structural levels". I wonder if it would be better to stick with the same "concepts" throughout the paper.
  - It mentions selling of body parts and blood. If this is a comparison to other organs, it should be made clearer.
  - Not clear the distinction between "Our review 32 corroborates the findings of previous studies, which have identified economic factors as

the primary drivers of kidney vending in India, Pakistan, Bangladesh, and Nepal" and ". Furthermore, some of the studies also described vulnerability to alleviate poverty as the reason for sales"

- Explicit Chenai as a city in India
- "Similarly, gendered views on the role of kidney played critical role in kidney selling". Not sure how this sentence adds to the previous long explanation of gender roles. Please, clarify.
- Spell out "HIC"
- Can you explain how the "proliferation of biotechnology within LMICs" ... contributes to the phenomenon of kidney selling?
- "The renewed techno-ignorance fundamentally, may have contributed to increase submission to advanced medical procedures including the exploitations" - this sentence reveals an issue at an individual level. This suggests that levels are intertwined. It is an obvious statement but the read might benefit from having clarity about it.
