# Supplementary material for: Why do people sell their kidneys? A thematic synthesis of qualitative evidence
Source: PLOS Glob Public Health. 2024 Mar 27;4(3):e0003015. doi: 10.1371/journal.pgph.0003015 (PMC10971689; doi:10.1371/journal.pgph.0003015)
Supplement: Attachment — Submitted filename: Manuscript Review PGPH-D-23-01387.docx [file pgph.0003015.s007.docx]

**Report on “Why do people sell their kidneys? A thematic synthesis of qualitative evidence”**

**Summary:**

This qualitative systematic review was conducted to explore the reasons for kidney selling around the globe. Literature related to kidney selling and its drivers was explored in three databases covering the period from 1987 to 2022 and a total of 15 articles were selected for the review, which underwent thematic analysis. Authors identified poverty and illiteracy as significant micro-level factors. Weak legal system, lacking stringent institutional policy, regulatory framework were identified as meso-level factors whereas social inequalities, corruption, organ shortage, insufficient health infrastructure have been mentioned as macro-level factors.

Overall, the paper is well-written and easy to follow, and the aims are well-formulated and clearly communicated. However, I have some concerns about some sections as well as some other less substantial questions and comments. My comments are ordered in two sections below: Major comments and minor comments.

**Major comments:**

| **Page/Line no.** | **Section** | **Manuscript** | **Comment** |
| --- | --- | --- | --- |
| Pg – 2,  L: 30-32 | Abstract | “The thematic analysis involved a critical approach to understanding the reasons for kidney selling by examining power disparities and social inequalities.” | Has this thematic analysis been done using any specific framework? If yes, the name of the analytical framework should be mentioned here. |
| Pg – 3,  L: 55-58 | Introduction | “However, the supply of kidneys has not been able to meet the growing demand. Kidneys are in high demand globally, with low- and middle-income countries (LMICs) being major suppliers. Poverty, vulnerability, and lack of awareness have been identified as factors that facilitate kidney selling.” | These sentences seem to state facts and make claims about the supply and demand for kidneys and the factors contributing to kidney selling. If these statements are intended to be presented as factual information, it is important to support them with appropriate references or sources to establish their credibility and accuracy. Without references, it's difficult to assess the validity of these claims. |
| Pg – 3  L: 61 - 63 | Introduction | “Kidney sellers are often unaware about the consequences of selling kidneys such as disabilities of various extent such as weakness, chronic kidney disease, and stigma.” | This statement also represents a fact here. Appropriate reference or source should be added here. |
| Pg – 3  L: 70 – 73 | Introduction | “Unfortunately, illegal organ trafficking has been a common occurrence in low-and middle-income countries (LMICs) which exploits vulnerable individuals facing desperate living conditions. Although organ trafficking has been condemned and deemed illegal, it remains persistent because of the lack of regulatory measures.” | These sentences denote the issue of illegal organ trafficking in low- and middle-income countries (LMICs) and suggest that it persists due to the lack of regulatory measures and seem to be facts. Appropriate reference(s) should be added here. |
|  | Methods |  | The contributions of three authors (BS, MS & BA) have been mentioned specifically. What were the roles and contributions of the rest of the authors in this study? The contribution of all the authors should be mentioned clearly. |
| Pg – 7,  L: 199-202 | Results | “The studies selected for final analysis showed considerable geographic variability: Asia-Pacific (India, Pakistan, The Philippines, Indonesia, Nepal), Europe (Moldova, The Netherlands), Middle East (Israel, Iran), North America (Canada), South America (Brazil), and Africa (South Africa).” | Figure 1 and Supplementary table 1 show that two studies from Bangladesh have also been included in this systematic review. However, “Bangladesh” seems missing from this list. |
| Pg – 1 | Results | Figure 1: Geographic coverage and themes derived from the places. | Specific themes for all the studies from different countries (including Bangladesh) have been mentioned here except Canada. Did the authors not find any specific theme for the study conducted in Canada? If not, this should be mentioned somewhere in the result section or in this figure. |
| Pg – 3  L: 2 - 4 | Results | “However, none of the participants were able to achieve financial freedom. Conversely, they were rather drawn into additional debt cycle and had poor health conditions which further restricted them to earn a living (2, 20, 31, 32).” | This section is particularly discussing about the participants from the included 15 studies in this systematic review. However, Reference no. 20 (Naqvi et al., 2007) is not one of the studies included in this systematic review. The authors should reconsider/reevaluate whether to include this study in the systematic review based on the significance of its findings and its alignment with all the inclusion criteria for this study. |
| Pg – 3  L: 5 - 7 | Results | “Participants also shared their willingness to donate kidneys; however, they were compensated with financial deals and other benefits such as child education. Some participants claimed that their deeds were just altruistic rooted in religious beliefs (17, 38). | This section is also discussing participants’ willingness for donating kidneys and these findings are from the included 15 studies in this systematic review. However, Reference no. 17 (Tong et al., 2012) is not one of the studies included in this systematic review. The authors should reconsider/reevaluate whether to include this study in the systematic review based on the significance of its findings and its alignment with all the inclusion criteria for this study or removing this reference from here if it is not relevant. |
|  | Results |  | Overall, the result section is good. The findings have been included for both individual and societal level. However, it seems that authors have prioritized describing or explaining the findings mostly from the Asian countries whereas some other studies from European, American, and African countries have been included in this systematic review. Similarities or variation of factors for kidney selling among these studies will be helpful for better understanding. |
| Pg – 6  L: 6 - 10 | Discussion | “Chennai also was an example for broader structural contributor for kidney selling. The convergence of wealthy and urban impoverished individuals comprised areas where the poor individuals experienced comparable deficits. As a compensation to poverty, the poor people felt motivated to sell their kidneys which was facilitated by Chennai’s advanced medical infrastructure for kidney transplantation.” | This statement seems like a fact described how Chennai was explained as an example for structural contributor to kidney selling. Appropriate reference or source should be cited here. |
| Pg – 6  L: 24 | Discussion | “In LMICs, a lot of HIC patients approach for cheap kidneys.” | Abbreviation of “HIC” was not mentioned earlier. Full form of HIC should be added here. |

**Minor comments:**

| **Page/Line no.** | **Section** | **Manuscript** | **Comment** |
| --- | --- | --- | --- |
| P – 5,  L: 120 | Methods |  | Has a new passage started here? If so, the space between the two paragraphs should be reformatted. |
| P – 6,  L: 170 | Methods | “…and the wealthiest members of society (24). We analyzed the situation…” | There is a double space after the period. |
| Pg – 1 | Results | Figure 1: Geographic coverage and themes derived from the places. | The page number may have been added incorrectly. Please review the document and ensure that the page numbers are accurate and properly formatted. Also, the line numbers should not be added to this page. |
| Pg – 1 | Results | Figure 2: Flowchart showing screening and inclusion of studies in the review | The page number may have been added incorrectly. Please review the document and ensure that the page numbers are accurate and properly formatted. Also, the line numbers should not be added to this page. |
| Pg – 2 | Results | Comprehensiveness of reporting | The page number may have been added incorrectly.  The Line numbers have been started from “1” again in this page. Incorrect page and line numbers should be corrected accordingly. |
| Pg – 2  L: 17 | Results | “A thematic analysis utilizing the lens of power relations and social inequities has been used to conduct this review (22) .” | An extra space has been added before the period. |
| Pg – 2  L: 27 | Results | “Many participants sold their kidneys due to lack of better economic conditions, such as jobs, basic…” | Has a new paragraph started here? If so, the space between the two paragraphs should be reformatted. |
| Pg – 3  L: 1 | Results |  | The page number may have been added incorrectly.  The Line numbers have been started from “1” again in this page. Incorrect page and line numbers should be corrected accordingly. |
| Pg – 3  L: 5  L: 8  L: 13 L: 31 | Results |  | It seems like new paragraphs have been started in these lines. Appropriate spacing should be done for the new paragraphs. |
| Pg – 4  L: 1 | Results |  | Page number and line numbers should be corrected. |
| Pg – 5  L: 1 | Results |  | Page number and line numbers should be corrected. |
| Pg – 6  L: 1 | Discussion |  | Page number and line numbers should be corrected. |
| Pg – 6  L: 13 | Discussion |  | If a new paragraph is starting from this line, spacing should be adjusted accordingly. |
| Pg – 7  L: 1 |  |  | Page number and line numbers should be corrected. |
| Pg – 8  L: 1 |  |  | Page number and line numbers should be corrected. |
