# Supplementary material for: Why do people sell their kidneys? A thematic synthesis of qualitative evidence
Source: PLOS Glob Public Health. 2024 Mar 27;4(3):e0003015. doi: 10.1371/journal.pgph.0003015 (PMC10971689; doi:10.1371/journal.pgph.0003015)
Supplement: Attachment — Submitted filename: Reviewers comments_PGPH.docx [file pgph.0003015.s008.docx]

**Why do people sell their kidneys? A thematic synthesis of qualitative evidence**

**Manuscript number: PGPH-D-23-01387**

**Reviewers Comments**

This is a very interesting systematic review (qualitative) in the field of public health. It describes why kidney trade occurs and how it is also affecting the health of most people in developing countries. The manuscript is well-written showing empirical evidence.

After review, there were no identifiable major issues, however the authors should address the following significant minor issues.

**Abstract**

It is very concise and includes all the needed sections.

**Introduction**

Line 89 and 90 sentences should be linked together to create a better understanding.

“…….. become more refined and minimally invasive. Including the medical technologies to …….”

**Materials and method**

**Search strategy**

1. Could the authors rename the supplementary Table 4 as Table 1 in order to ensure consistency in the manuscript? The numbering of the Tables should be consistent in order of appearance in the manuscript. Thus, Table 1 should be referenced before Table 2 and in that order. However, the supposed supplementary Table 4 does not look like a Table. So critical modifications should be made on the presentation of the Tables.

**Study selection**

1. Line 127 sentence should be revised. “ All studies studies were included that offered qualitative evidence on selling kidneys for commercial reasons including motivations to sell their kidneys.”
2. Did the study include “Preprint articles” from 1987 to 2022? Kindly spell it out in the inclusion and exclusion criteria.

**Data extraction and quality assessment**

1. COREQ should be defined (written in full) at the base of Table 2.

**Data synthesis and analysis**

1. Kindly modify the last sentence under ‘Data synthesis and analysis’. Thus, “All of the authors agreed that the final themes which are the basis of the results in this review”

**Results**

**Study selection**

1. The authors should rename Figure 2 as Figure 1 and vice versa for consistency on how they appear or are referenced in the manuscript.

**Discussion**

1. The country where Chennai is located should be mentioned for more comprehension.
2. HIC must first be defined before using the abbreviation.

**References**

1. Reference #28, some of the wordings are capitalized while others are written in small letters. Check through other references and correct them as well, except recognized abbreviated words.
2. Reference #43 (Thus, “Kaiju I. मिर्गौला फे र्न ‘र्क्कली’ र्ाता. Annapurna Post 2018”) should be cited in English and well written
