# Supplementary material for: Why do people sell their kidneys? A thematic synthesis of qualitative evidence
Source: PLOS Glob Public Health. 2024 Mar 27;4(3):e0003015. doi: 10.1371/journal.pgph.0003015 (PMC10971689; doi:10.1371/journal.pgph.0003015)
Supplement: Attachment — Submitted filename: Response to reviewers.docx [file pgph.0003015.s009.docx]

**Why do people sell their kidneys? A thematic synthesis of qualitative evidence**

**Manuscript number: PGPH-D-23-01387**

**Reviewers Comments**

**Reviewer#1**

I greatly enjoyed reading the manuscript, which sheds light on a relevant public health issue that requires being properly addressed by authorities worldwide. Appendices are very rich in information.

**AUTHORS:** Thank you for the feedback.

Some comments and suggestions:

**Row 39 - the notion of goods for pleasure. I would suggest thinking about the notion as not necessarily reflects the purpose of the acquired good. For example, a mobile phone or a motorbike could be an economic means. People might consider it as a “necessity” and not as something superfluous.**

**AUTHORS:** Thank you for the feedback. While you may be right, these are impoverished communities where day to day living is very much dependent on cash money. In my own observations (owing to ethnographical study)(1), I saw the kidney sellers misusing revenues from kidney selling spent on luxury items such as motorbikes, mobile phones, TV and refrigerators. Therefore, these items may have been bought without much fore-thinking about the saving and indeed does sound absurd, nonetheless that has been the reality in these communities.

**Row 41 - the word “Ironically” doesn’t express, at least in my opinion, the tragedy of doing that.**

**AUTHORS:** Thank you for the feedback. In contrast to how one would expect that they would learn from their own kidney selling, and prevent such acts in the community, kidney sellers later turned to be the brokers and you are right, because they do not have any other options to support their livelihoods. We have changed the word, ‘ironically’ to ‘surprisingly’.

**The introduction does not clearly state the unethical aspect of selling organs. For example, row 75 mentions “policies guiding kidney selling”. Is it legal in any country? I understand Iran is the only country that allows it. WHO has issued different condemnations of the financial transactions of organs. For instance, the 44th World Health Assembly.**

**AUTHORS:** Thank you for the feedback. We have added ethical aspects of kidney selling and reads as follows:

Nonetheless, ethical arguments of kidney selling are unaddressed by the legal frameworks. Even if legal frameworks are meant to safeguard the exploitation involved in kidney selling, the kidney selling amongst the vulnerable poor compromises the central tenet of autonomy in consent process. The consent itself for vulnerable donors is affected by undue inducement by incentives involved in transaction. At the same time, recipients are vulnerable because their opportunity to living will be compromised if they do not accept the donated organs from anyone including vulnerable donors. For both donors and recipients, the nature and degree of vulnerability tends to cloud the ethical principles of consent process. In addition, allowing selling of organs from mostly poor and vulnerable donors is likely to promote inequity as most of the buyers are privileged to afford while the poor may succumb to inability to buy an organ. Nonetheless, viewed from the fact that buyers have equal rights to afford organs for their living, it naturally selects those who are able to leaving poor in need of organs further vulnerable (2).

**Rows 100-102: I would add there are risks of acquiring infectious diseases such as Hepatitis and HIV.**

**AUTHORS:** Thank you for the feedback. We have added the statement as suggested.

**The readers will benefit from a clear definition of kidney selling in the Introduction. I mention that because the introduction reviews many different modalities (donation from living and death). Is kidney selling equivalent to kidney trafficking? How it differs?**

**AUTHORS:** Thank you for the feedback. We have added the definition of kidney selling and kidney trafficking in the introduction section as follows:

Kidney selling could solely be an act initiated by the kidney sellers to an institution or a person without necessarily involvement of a broker. Kidney trafficking whereas can be an act of exploitation, coercion and inducement to sell to an institution or a person with involvement of a broker.

**On the PRISMA graph, it says “Records excluded through title”. However, in the text, it explains that the initial screening goes beyond that. I would suggest changing it to “Records excluded through initial screening”.**

**AUTHORS:** Thank you for the feedback. Revised as suggested.

**On the analysis of the comprehensiveness of reporting, I wonder if there should be a discussion or comparison between those who report few items and those who report more items. Does it matter? Does it reflect quality?**

**AUTHORS:** Thank you for the feedback. In our opinion COREQ guideline outlines the framework of reporting and does not necessarily have impact on the quality. As some of the articles that did not report based on the COREQ guideline were able to offer rich details and thus the quality.

**About lens: In the Data synthesis and analysis (row 164ss) says “The thematic analysis took a critical approach to understand the reasons for kidney selling by examining power disparities and social inequalities” and later “We used the critical medical anthropology (CMA) theoretical lens”. On the results it mentions the “lens of power relations and social in- equities”, and on the abstract “The thematic analysis involved a critical approach to understanding”. I am a bit confused if there are differences between CMA and critical approach and power relations and social in- equalities.**

**AUTHORS:** Thank you for the feedback. We acknowledge the heterogeneity in our reporting. It should have been the CMA. We have revised the document to make it consistent throughout the document.

Results:

**individual level: “poverty was the major reason for kidney” and “desperation to alleviate poverty” seem similar to me.**

**“Many participants sold their kidneys due to lack of better economic conditions”. Do you mean lack of economic means such as jobs or regular income? If not, what is the difference between “poverty” and “lack of better economic conditions”?**

**AUTHORS:** Thank you for the feedback. We tried to delineate the differences between the state of being poor (poverty) and the attitude towards alleviating the poverty status. There are individuals who out of poverty sell their organs, and others who are motivated to broker kidney selling among their peers and neighbors. We have added the following statement to clarify that reads as follows:

Some kidney sellers were vulnerable to sell their kidney because of their state of poverty, while others tended to broker kidney selling in the community to alleviate their level of poverty.

**I think these paragraphs will benefit the reader if they are better	organised. I have tried to identify the main topics per paragraph	and it becomes difficult because it looks repetitive. I am sure the information is quite rich and relevant. So, for example (you don’t have to follow it), organise the paragraphs around topics: poverty, payment of debts, buying goods, altruism, etc.**

**The authors also report some consequences of selling kidneys such as “no economic freedom, poor health, etc”. That should go in a different paragraph to make the reading experience clear.**

**The same comment about organising the information applies to the meso and macro levels.**

**AUTHORS:** Thank you for the feedback. We have revised based on your suggestions.

Societal level:

**explain social conformism;**

**“Some of the participants sold their kidneys due to ignorance and unawareness regarding the function of kidneys”. This seems to belong to the individual level. I understand that brokers use that, but it would also be good to highlight it in the individual-level section.**

**AUTHORS:** Thank you for the feedback. We agree with you that this theme crosscuts at an individual and a societal level. We have revised statement to reflect this and reads as follows:

Conformism has been understood as a social dynamic reflecting the social cohesiveness, nonetheless, it’s role and impacts are also affected at an individual level. For instance, a person may act against the social norms of cohesiveness and conformity.

The section is called “Societal level” and on row 11 it mentions “intermediate and macro level”. Is this a different section?

**Figure 3 is the author’s own. Which author? If this is your contribution, it should be included as a section and explained as a synthesis of the reviews or similar. If you could map back to the original papers that would give robustness to the theoretical contribution.**

**AUTHORS:** Thank you for the question. The statement meant to explain authors’ contribution in synthesizing the diagram from this review. We have revised the figure legend to explain this and reads as follows:

**Figure 3**: Factors influencing kidney selling in Nepal. This figure is author’s original work based on this review describing...............

Discussion:

**I find difficult to distinguish between the results and the discussion sections. You could benefit from this “h**[**ttps://plos.org/resource/**](https://plos.org/resource/how-to-write-conclusions/) [**how-to-write-conclusions/”**](https://plos.org/resource/how-to-write-conclusions/)

**The fact that Iran has a “legal” structure of selling kidneys should be accounted for in the analysis, for example, to compare with other countries in the discussion.**

Mentions “structural levels”. I wonder if it would be better to stick with the same “concepts” throughout the paper.

It mentions selling of body parts and blood. If this is a comparison to other organs, it should be made clearer.

Not clear the distinction between “Our review 32 corroborates the findings of previous studies, which have identified economic factors as the primary drivers of kidney vending in India, Pakistan, Bangladesh, and Nepal” and “. Furthermore, some of the studies also described vulnerability to alleviate poverty as the reason for sales”

Explicit Chenai as a city in India

**AUTHORS:** Thank you for the feedback. As above, we have identified two main motivations for kidney selling. One that poverty itself propels kidney selling, and other that people in poor socioeconomic categories (not necessarily extremely poor) who are immersed in debt and have familial obligations such as dowry, tend to sell kidneys.

**“Similarly, gendered views on the role of kidney played critical role in kidney selling”. Not sure how this sentence adds to the previous long explanation of gender roles. Please, clarify.**

Spell out “HIC”

**AUTHORS:** Thank you for the suggestions.

Can you explain how the “proliferation of biotechnology within LMICs” . . . contributes to the phenomenon of kidney selling?

“The renewed techno-ignorance fundamentally, may have contributed to increase submission to advanced medical procedures including the exploitations” - this sentence reveals an issue at an individual level. This suggests that levels are intertwined. It is an obvious statement	but the read might benefit from having clarity about it.

**AUTHORS:** Thank you for the feedback. We have revised and added the content as required.

**Reviewer #2**

This is a very interesting systematic review (qualitative) in the field of public health. It describes why kidney trade occurs and how it is also affecting the health of most people in developing countries. The manuscript is well-written showing empirical evidence.

After review, there were no identifiable major issues, however the authors should address the following significant minor issues.

**Abstract**

It is very concise and includes all the needed sections.

**Introduction**

**Line 89 and 90 sentences should be linked together to create a better understanding.**

**“…….. become more refined and minimally invasive. Including the medical technologies to …….”**

**AUTHORS:** Thank you for the feedback. We have revised the statement as suggested and reads as follows:

The surgical techniques used in kidney transplantation have become more refined and minimally invasive. Increasingly, modern medical technologies have aided smooth transplantation thus promoting the kidney transplantation.

**Materials and method**

**Search strategy**

1. Could the authors rename the supplementary Table 4 as Table 1 in order to ensure consistency in the manuscript? The numbering of the Tables should be consistent in order of appearance in the manuscript. Thus, Table 1 should be referenced before Table 2 and in that order. However, the supposed supplementary Table 4 does not look like a Table. So critical modifications should be made on the presentation of the Tables.

**AUTHORS:** Thank you for the feedback. We have adjusted it accordingly.

**Study selection**

1. Line 127 sentence should be revised. “ All studies studies were included that offered qualitative evidence on selling kidneys for commercial reasons including motivations to sell their kidneys.”
2. Did the study include “Preprint articles” from 1987 to 2022? Kindly spell it out in the inclusion and exclusion criteria.

**AUTHORS:** Thank you for the feedback. We have revised it.

**Data extraction and quality assessment**

1. COREQ should be defined (written in full) at the base of Table 2.

**AUTHORS:** Thank you for the feedback. We have explained COREQ in Data extraction and quality assessment. In the Line ......

**Data synthesis and analysis**

1. Kindly modify the last sentence under ‘Data synthesis and analysis’. Thus, “All of the authors agreed that the final themes which are the basis of the results in this review”

**AUTHORS:** Thank you for the feedback. Revised sentence reads as follows:

“All of the authors agreed on the final themes which are the basis of the results in this review”

**Results**

**Study selection**

1. The authors should rename Figure 2 as Figure 1 and vice versa for consistency on how they appear or are referenced in the manuscript.

**AUTHORS:** Thank you for the feedback. We have revised it.

**Discussion**

1. The country where Chennai is located should be mentioned for more comprehension.
2. HIC must first be defined before using the abbreviation.

**AUTHORS:** Thank you for the feedback. We have revised it.

**References**

1. Reference #28, some of the wordings are capitalized while others are written in small letters. Check through other references and correct them as well, except recognized abbreviated words.
2. Reference #43 (Thus, “Kaiju I. मिर्गौला फे र्न ‘र्क्कली’ र्ाता. Annapurna Post 2018”) should be cited in English and well written

**AUTHORS:** Thank you for the feedback. We have revised it.

**Reviewer#3**

**Report on “Why do people sell their kidneys? A thematic synthesis of qualitative evidence”**

**Summary:**

This qualitative systematic review was conducted to explore the reasons for kidney selling around the globe. Literature related to kidney selling and its drivers was explored in three databases covering the period from 1987 to 2022 and a total of 15 articles were selected for the review, which underwent thematic analysis. Authors identified poverty and illiteracy as significant micro-level factors. Weak legal system, lacking stringent institutional policy, regulatory framework were identified as meso-level factors whereas social inequalities, corruption, organ shortage, insufficient health infrastructure have been mentioned as macro-level factors.

Overall, the paper is well-written and easy to follow, and the aims are well-formulated and clearly communicated. However, I have some concerns about some sections as well as some other less substantial questions and comments. My comments are ordered in two sections below: Major comments and minor comments.

**AUTHORS:** Thank you for the feedback. We have revised it and responded.

**Major comments:**

| **Page/Line no.** | **Section** | **Manuscript** | **Comment** | **Authors’ responses** |
| --- | --- | --- | --- | --- |
| Pg – 2,  L: 30-32 | Abstract | “The thematic analysis involved a critical approach to understanding the reasons for kidney selling by examining power disparities and social inequalities.” | Has this thematic analysis been done using any specific framework? If yes, the name of the analytical framework should be mentioned here. | We have used the theoretical approach of Critical Medical Anthropology to explain it. |
| Pg – 3,  L: 55-58 | Introduction | “However, the supply of kidneys has not been able to meet the growing demand. Kidneys are in high demand globally, with low- and middle-income countries (LMICs) being major suppliers. Poverty, vulnerability, and lack of awareness have been identified as factors that facilitate kidney selling.” | These sentences seem to state facts and make claims about the supply and demand for kidneys and the factors contributing to kidney selling. If these statements are intended to be presented as factual information, it is important to support them with appropriate references or sources to establish their credibility and accuracy. Without references, it's difficult to assess the validity of these claims. | Thank you and we have realized it and cited it as well. |
| Pg – 3  L: 61 - 63 | Introduction | “Kidney sellers are often unaware about the consequences of selling kidneys such as disabilities of various extent such as weakness, chronic kidney disease, and stigma.” | This statement also represents a fact here. Appropriate reference or source should be added here. | Thank you and we have realized it and cited it as well. |
| Pg – 3  L: 70 – 73 | Introduction | “Unfortunately, illegal organ trafficking has been a common occurrence in low-and middle-income countries (LMICs) which exploits vulnerable individuals facing desperate living conditions. Although organ trafficking has been condemned and deemed illegal, it remains persistent because of the lack of regulatory measures.” | These sentences denote the issue of illegal organ trafficking in low- and middle-income countries (LMICs) and suggest that it persists due to the lack of regulatory measures and seem to be facts. Appropriate reference(s) should be added here. | Thank you and we have realized it and cited it as well. |
|  | Methods |  | The contributions of three authors (BS, MS & BA) have been mentioned specifically. What were the roles and contributions of the rest of the authors in this study? The contribution of all the authors should be mentioned clearly. | All other authors offered inputs and reviews in the review outcomes. |
| Pg – 7,  L: 199-202 | Results | “The studies selected for final analysis showed considerable geographic variability: Asia-Pacific (India, Pakistan, The Philippines, Indonesia, Nepal), Europe (Moldova, The Netherlands), Middle East (Israel, Iran), North America (Canada), South America (Brazil), and Africa (South Africa).” | Figure 1 and Supplementary table 1 show that two studies from Bangladesh have also been included in this systematic review. However, “Bangladesh” seems missing from this list. | Thank you for pointing out the country Bangladesh. We have added it. |
| Pg – 1 | Results | Figure 1: Geographic coverage and themes derived from the places. | Specific themes for all the studies from different countries (including Bangladesh) have been mentioned here except Canada. Did the authors not find any specific theme for the study conducted in Canada? If not, this should be mentioned somewhere in the result section or in this figure. | Because there was no mention of selling, but on donors’ perspective. It was not under our central theme. |
| Pg – 3  L: 2 - 4 | Results | “However, none of the participants were able to achieve financial freedom. Conversely, they were rather drawn into additional debt cycle and had poor health conditions which further restricted them to earn a living (2, 20, 31, 32).” | This section is particularly discussing about the participants from the included 15 studies in this systematic review. However, Reference no. 20 (Naqvi et al., 2007) is not one of the studies included in this systematic review. The authors should reconsider/reevaluate whether to include this study in the systematic review based on the significance of its findings and its alignment with all the inclusion criteria for this study. | Thank you for feedback. We have removed the reference. |
| Pg – 3  L: 5 - 7 | Results | “Participants also shared their willingness to donate kidneys; however, they were compensated with financial deals and other benefits such as child education. Some participants claimed that their deeds were just altruistic rooted in religious beliefs (17, 38). | This section is also discussing participants’ willingness for donating kidneys and these findings are from the included 15 studies in this systematic review. However, Reference no. 17 (Tong et al., 2012) is not one of the studies included in this systematic review. The authors should reconsider/reevaluate whether to include this study in the systematic review based on the significance of its findings and its alignment with all the inclusion criteria for this study or removing this reference from here if it is not relevant. | We have corrected. |
|  | Results |  | Overall, the result section is good. The findings have been included for both individual and societal level. However, it seems that authors have prioritized describing or explaining the findings mostly from the Asian countries whereas some other studies from European, American, and African countries have been included in this systematic review. Similarities or variation of factors for kidney selling among these studies will be helpful for better understanding. | Thank you |
| Pg – 6  L: 6 - 10 | Discussion | “Chennai also was an example for broader structural contributor for kidney selling. The convergence of wealthy and urban impoverished individuals comprised areas where the poor individuals experienced comparable deficits. As a compensation to poverty, the poor people felt motivated to sell their kidneys which was facilitated by Chennai’s advanced medical infrastructure for kidney transplantation.” | This statement seems like a fact described how Chennai was explained as an example for structural contributor to kidney selling. Appropriate reference or source should be cited here. | Thank you for the feedback and cited. |
| Pg – 6  L: 24 | Discussion | “In LMICs, a lot of HIC patients approach for cheap kidneys.” | Abbreviation of “HIC” was not mentioned earlier. Full form of HIC should be added here. | Thank you |

**Minor comments:**

| **Page/Line no.** | **Section** | **Manuscript** | **Comment** | **Authors’ responses** |
| --- | --- | --- | --- | --- |
| P – 5,  L: 120 | Methods |  | Has a new passage started here? If so, the space between the two paragraphs should be reformatted. | Thank you for the feedback. Edited. |
| P – 6,  L: 170 | Methods | “…and the wealthiest members of society (24). We analyzed the situation…” | There is a double space after the period. | Thank you for the feedback. Edited. |
| Pg – 1 | Results | Figure 1: Geographic coverage and themes derived from the places. | The page number may have been added incorrectly. Please review the document and ensure that the page numbers are accurate and properly formatted. Also, the line numbers should not be added to this page. | Thank you for the feedback. Edited. |
| Pg – 1 | Results | Figure 2: Flowchart showing screening and inclusion of studies in the review | The page number may have been added incorrectly. Please review the document and ensure that the page numbers are accurate and properly formatted. Also, the line numbers should not be added to this page. | Thank you for the feedback. Edited. |
| Pg – 2 | Results | Comprehensiveness of reporting | The page number may have been added incorrectly.  The Line numbers have been started from “1” again in this page. Incorrect page and line numbers should be corrected accordingly. | Thank you for the feedback. Edited. |
| Pg – 2  L: 17 | Results | “A thematic analysis utilizing the lens of power relations and social inequities has been used to conduct this review (22) .” | An extra space has been added before the period. | Thank you for the feedback. Edited. |
| Pg – 2  L: 27 | Results | “Many participants sold their kidneys due to lack of better economic conditions, such as jobs, basic…” | Has a new paragraph started here? If so, the space between the two paragraphs should be reformatted. | Thank you for the feedback. Edited. |
| Pg – 3  L: 1 | Results |  | The page number may have been added incorrectly.  The Line numbers have been started from “1” again in this page. Incorrect page and line numbers should be corrected accordingly. | Thank you for the feedback. Edited. |
| Pg – 3  L: 5  L: 8  L: 13 L: 31 | Results |  | It seems like new paragraphs have been started in these lines. Appropriate spacing should be done for the new paragraphs. | Thank you for the feedback. Edited. |
| Pg – 4  L: 1 | Results |  | Page number and line numbers should be corrected. | Thank you for the feedback. Edited. |
| Pg – 5  L: 1 | Results |  | Page number and line numbers should be corrected. | Thank you for the feedback. Edited. |
| Pg – 6  L: 1 | Discussion |  | Page number and line numbers should be corrected. | Thank you for the feedback. Edited. |
| Pg – 6  L: 13 | Discussion |  | If a new paragraph is starting from this line, spacing should be adjusted accordingly. | Thank you for the feedback. Edited. |
| Pg – 7  L: 1 |  |  | Page number and line numbers should be corrected. | Thank you for the feedback. Edited. |
| Pg – 8  L: 1 |  |  | Page number and line numbers should be corrected. | Thank you for the feedback. Edited. |

1.	Shrestha B, Adhikari B, Shrestha M, Poudel A, Shrestha B, Sunuwar DR, et al. ‘The broker also told me that I will not have problems after selling because we have two and we can survive on one kidney’: Findings from an ethnographic study of a village with one kidney in Central Nepal. PLOS Global Public Health. 2022;2(10):e0000585.

2.	Kishore RR. Human organs, scarcities, and sale: morality revisited. Journal of medical ethics. 2005;31(6):362-5.
